# Non-Orthogonality of QAM and Sunflower-like Modulated Coherent-State Signals

**DOI:** 10.3390/e27010030

**Published:** 2025-01-01

**Authors:** Kentaro Kato

**Affiliations:** Quantum ICT Research Institute, Tamagawa University, Tokyo 194-8610, Japan; kkatop@lab.tamagawa.ac.jp

**Keywords:** non-orthogonality, detection probability, square-root measurement, signal constellation

## Abstract

The limitations of cloning and discriminating quantum states are related to the non-orthogonality of the states. Hence, understanding the collective features of quantum states is essential for the future development of quantum communications technology. This paper investigates the non-orthogonality of different coherent-state signal constellations used in quantum communications, namely phase-shift keying (PSK), quadrature-amplitude modulation (QAM), and a newly defined signal named the sunflower-like (SUN) coherent-state signal. The non-orthogonality index (NOI) and the average probability of correct detection (detection probability) are numerically computed. Results show that PSK NOI increases faster than QAM and SUN as the number of signals increases for a given number of signal photons. QAM and SUN exhibit similar NOI and detection probability, behaving similarly to randomly generated signals for a larger number of signals. Approximation formulas are provided for the detection probability as a function of NOI for each signal type. While similar to QAM, SUN signal offers potential advantages for applications requiring uniform signal-space distribution. The findings provide valuable insights for designing useful quantum signal constellations.

## 1. Introduction

The limitations of cloning [1,2,3] and discriminating [4,5] quantum states are related to the non-orthogonality of the states. The author introduced the non-orthogonality index (NOI) in reference [6] to quantify the non-orthogonality of a collection of pure quantum states, a crucial step toward our understanding of the collective feature of quantum states used in current and future quantum communications technology.

NOI indicates a value between zero and one for a given collection of pure quantum states. When NOI is zero, it means that the quantum states in a given collection are orthogonal, and thus, complete cloning and error-free discrimination of the states is possible. Such an error-free scenario is classical, without quantum mechanical properties. Conversely, if NOI approaches one, making the states nearly indistinguishable, it becomes impossible to identify the quantum mechanical states, even with a quantum mechanically designed measurement. Thus, our interest lies in scenarios where NOI is relatively high rather than relatively low.

Understanding the properties of quantum-state collections is essential for analyzing and designing information and communication systems that process information using quantum mechanical effects, and it provides a practical guideline for designing optical signals in the context of quantum communications, including quantum cryptographic applications [7,8,9]. In reference [6], the author investigated the non-orthogonality of phase-shift keying (PSK) coherent-state signals and studied its relationship with the channel capacity. Thus, the linking of physical indices, such as non-orthogonality, with evaluation factors for technological potential, such as channel capacity, is essential for advancing quantum communications technology.

This paper investigates the NOI of quadrature amplitude modulation (QAM) coherent-state signal and a newly defined signal. QAM is a modulation format that arranges signal points in a rectangular pattern in the phase space of light. The new type of signal is defined using the golden ratio for angles [10], and the signal points are placed in the phase space in a way similar to the arrangement of sunflower seeds. In addition to investigating the non-orthogonality, we will also explore the detection performance of each modulation format. We will calculate the average probability of correct detection (detection probability), supposing a quantum mechanically designed measurement called the square-root measurement (SRM) [11,12,13].

This paper is organized as follows: Section 2 briefly summarizes NOI and the detection probability, which are the indices to be calculated. Section 3 describes PSK, QAM, and the new signal. Section 4 presents the calculation results for the index of each signal. Section 4 also calculates the randomly generated signals for reference. Finally, we discuss the results in Section 5 and provide conclusions in Section 6.

## 2. Non-Orthogonality of a Collection of Pure Quantum States

### 2.1. Non-Orthogonality Index

Consider a collection of *M* linearly independent quantum states, $S=\{|\psi_{m}\rangle :m=1,2,\ldots ,M\}$. For this collection of pure quantum states, the non-orthogonal index (NOI) is given by

(1) $$NOI\left(S\right)=\frac{1}{2(M-\sqrt{M})}\sum_{m=1}^{M}{\left(1-\sqrt{\lambda_{m}}\right)}^{2},$$

where $\lambda_{m}$ is the *m*th eigenvalue of the Gram matrix $G=[\langle \psi_{m}|\psi_{m^{\prime }}\rangle ]$ [6]. This index serves to quantify the degree of non-orthogonality exhibited collectively by quantum states.

### 2.2. Square-Root Measurement

Let us describe the square-root measurement (SRM) for S [11,12,13]. The positive operator-valued measure $\left\{{\hat{\Pi }}_{m}^{\mathrm{SRM}}:m=1,2,\ldots ,M\right\}$ representing SRM for S is defined by

(2) $${\hat{\Pi }}_{m}^{\mathrm{SRM}}={\hat{G}}^{-1/2}\left|\psi_{m}\rangle \langle \psi_{m}\right|{\hat{G}}^{-1/2},\;m=1,2,\ldots ,M;\;\hat{G}=\sum_{m=1}^{M}|\psi_{m}\rangle \langle \psi_{m}|.$$

The probability of detecting the $m^{\prime }$th state by SRM when the *m*th state was true is calculated as $P\left(m^{\prime }|m\right)=\langle \psi_{m}|{\hat{\Pi }}_{m^{\prime }}^{\mathrm{SRM}}|\psi_{m}\rangle$. Therefore, the average probability of correct detection ${\bar{P}}_{c}^{\mathrm{SRM}}$ and the average probability of error ${\bar{P}}_{e}^{\mathrm{SRM}}$ are expressed as

(3) $${\bar{P}}_{c}^{\mathrm{SRM}}=\frac{1}{M}\sum_{m=1}^{M}{\left|\langle \psi_{m}|{\hat{G}}^{-1/2}|\psi_{m}\rangle \right|}^{2}=\frac{1}{M}\sum_{m=1}^{M}{\left|G_{mm}^{1/2}\right|}^{2},\;{\bar{P}}_{e}^{\mathrm{SRM}}=1-{\bar{P}}_{c}^{\mathrm{SRM}}.$$

Calculating these probabilities requires the square root of the Gram matrix, so the computational cost is higher than the simple eigenvalue calculation.

## 3. Signal Constellation

A collection of quantum mechanical states of light means a modulated optical signal in the context of quantum communications. This section will describe three types of signals consisting of coherent states of light. In particular, one of them is a novel type using the so-called golden angle.

The coherent state with complex amplitude α is defined as the eigenstate of the annihilation operator $\hat{a}$ with eigenvalue α, i.e., $\hat{a}{|\alpha \rangle }_{\mathrm{coh}}=\alpha {|\alpha \rangle }_{\mathrm{coh}}$, and the average number of photons of the state ${|\alpha \rangle }_{\mathrm{coh}}$ is n¯=〈cohα|n^|α〉coh=|α|2, where $\hat{n}={\hat{a}}^{†}\hat{a}$ is the number operator.

### 3.1. Phase Shift Keying Coherent State Signal

**Figure 1:**
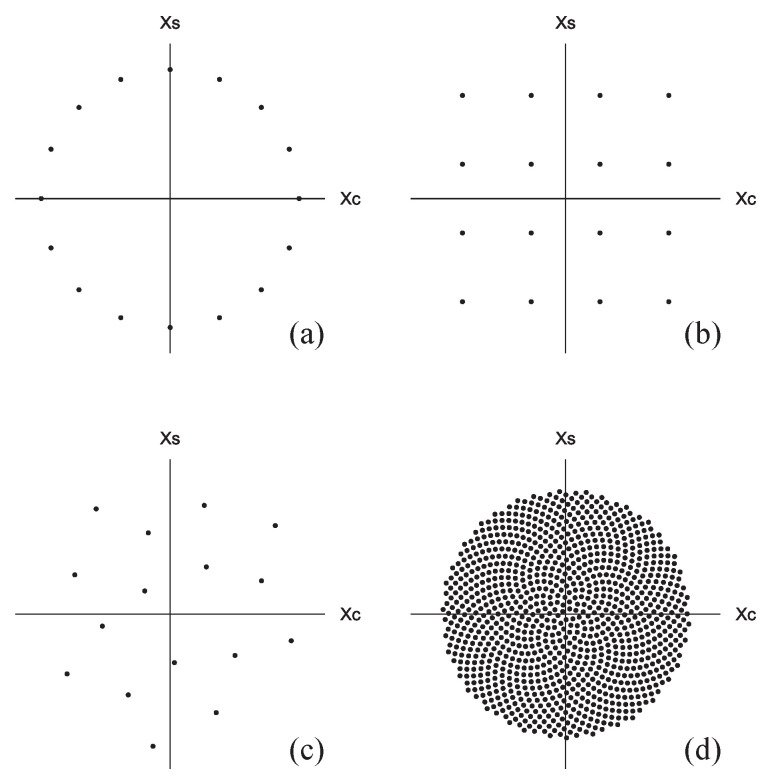
Examples of signal constellations. (**a**) 16-PSK. (**b**) 16-QAM. (**c**) 16-SUN. (**d**) 1024-SUN.

*M*-ary PSK coherent state signal is defined by

(4) $$S^{\mathrm{PSK}}=\left\{|\alpha_{0}\exp\left[\frac{2m\pi j}{M}\right]\rangle_{\mathrm{coh}}:m=0,1,\ldots ,M-1\right\},$$

where $j=\sqrt{-1}$ and $\alpha_{0}>0$. The signal constellation of 16-PSK is illustrated in Figure 1a, where ${\hat{x}}_{c}=\left({\hat{a}}^{†}+\hat{a}\right)/2$ and ${\hat{x}}_{s}=\left({\hat{a}}^{†}-\hat{a}\right)/2j$. The average number of photons for PSK is ${\bar{n}}_{s}=\alpha_{0}^{2}$, where each signal was assumed to be equiprobable, P(m)=1/M.

The eigenvalues of the Gram matrix for *M*-PSK are analytically given by

(5) $$\lambda_{m}=\sum_{\ell =1}^{M}A_{\left(1,\ell \right)}cos\left[\Theta_{\left(1,\ell \right)}-\frac{2\pi m(\ell -1)}{M}\right],$$

where

(6) $$A_{\left(1,\ell \right)}=\exp\left[-2\alpha_{0}^{2}\sin^{2}\left[\frac{\pi (\ell -1)}{M}\right]\right],\;\Theta_{\left(1,\ell \right)}=\alpha_{0}^{2}\sin\left[\frac{2\pi (\ell -1)}{M}\right].$$

Substituting these eigenvalues into Equation (1), the NOI of *M*-PSK can be obtained analytically [14]. Furthermore, the average probability of correct detection by SRM for PSK is given by

(7) $${\bar{P}}_{c}^{\mathrm{PSK},\mathrm{SRM}}=\frac{1}{M^{2}}{\left(\sum_{m=1}^{M}\sqrt{\lambda_{m}}\right)}^{2},$$

where each state of the signal is assumed to be equiprobable [14]. Since the states in $S^{\mathrm{PSK}}$ are symmetric (or homogeneous), $\left\{{\hat{\Pi }}_{m}^{\mathrm{SRM}}\right\}$ is optimal in the error probability criterion [15,16].

### 3.2. Quadrature Amplitude Modulation (QAM) Coherent State Signal

Suppose *M* can be written in $M=L^{2}$ by an integer L≥2, and define the index set

(8) Ω={−(L−1)+2(k−1):k=1,2,…,L}.

Using this index set, *M*-ary quadrature amplitude modulated (QAM) coherent state signal is given as $S^{\mathrm{qam}}={\{|\beta_{0}\left(x+jy\right)\rangle }_{\mathrm{coh}}:\left(x,y\right)\in \Omega^{2}\}$, where $\beta_{0}>0$ [14].

The average number of signal photons ${\bar{n}}_{s}$ for *M*-QAM is

(9) $${\bar{n}}_{s}=\frac{2\left(M-1\right)\beta_{0}^{2}}{3}.$$

Typical values for QAM are shown in Table 1, together with other cases.

It is difficult to obtain the eigenvalues and the square root of the Gram matrix for QAM analytically, so we compute them numerically.

### 3.3. ‘Sunflower’ Coherent State Signal

Here, we define a new signal. Since it is constructed to resemble the arrangement of sunflower seeds, we will call it the ‘sunflower’ signal and abbreviate it to SUN.

*M*-ary SUN coherent state signal $S^{\mathrm{sun}}=\left\{|\gamma_{0}\left(x_{m}+jy_{m}\right)\rangle_{\mathrm{coh}}:m=1,2,\ldots ,M\right\}$ is defined by

(10) $$x_{m}=\sqrt{m}\cos\left[m\Phi \right]\;\mathrm{and}\;y_{m}=\sqrt{m}\sin\left[m\Phi \right],$$

where $\gamma_{0}>0$ and $\Phi =\left(3-\sqrt{5}\right)\pi ∼2.39996\;\left[\mathrm{rad}\right]∼137.508\;\left[\deg\right]$ is the golden angle [10], and Equation (10) has been inspired by the formula found in reference [17]. 16-SUN and 1024-SUN are illustrated, respectively, in Figure 1c,d. If *M* is sufficiently large, as in the case of Figure 1(d), the signal points will be arranged almost uniformly within a circle.

The main difference between SUN and QAM is that while QAM has the $M=L^{2}$ restriction on the number of signals, SUN can be realized with any number of signals. Besides the cryptographic applications mentioned in the introduction, SUN also has potential as a base format for probabilistic shaping and geometric shaping in digital coherent optical communication systems [18,19].

The average number of signal photons ${\bar{n}}_{s}$ for *M*-SUN is

(11) $${\bar{n}}_{s}=\frac{\left(M+1\right)\gamma_{0}^{2}}{2}.$$

Typical values for SUN are shown in Table 1, together with the cases of PSK and QAM.

For SUN, the eigenvalues and the square root of the Gram matrix are computed numerically, as in the case of QAM.

## 4. Non-Orthogonality and Detection Probability for PSK, QAM, and SUN

In this section, we examine the non-orthogonality index NOI and the detection probability ${\bar{P}}_{c}$. For PSK and SUN, we calculate NOI and ${\bar{P}}_{c}$ for each *M* from 16 to 4096. For QAM, the calculations were performed in the range of *M* from 16 to 4096, considering the restriction of $M=L^{2}$.

For reference, we also consider the case of a ‘random’ *M*-ary coherent state signal, which consists of *M* coherent states chosen at random. For *M* values from 16 to 1024, 50 samples are generated for each *M*. When *M* exceeds 1024, calculations are performed for several values of *M*, precisely M= 1176, 1351, 1552, 1783, 2048, 2353, 2702, 3104, and 4096. Being selective with a large *M* is due to the computational time.

In all cases, we set the average number of photons ${\bar{n}}_{s}$ in increments of 10, ranging from 10 to 100 photons.

The calculation results are shown in the next subsections. All figures indicate PSK, QAM, SUN, and the random signals by blue, black, red, and yellow dots, respectively.

### 4.1. NOI vs. *M*

**Figure 2:**
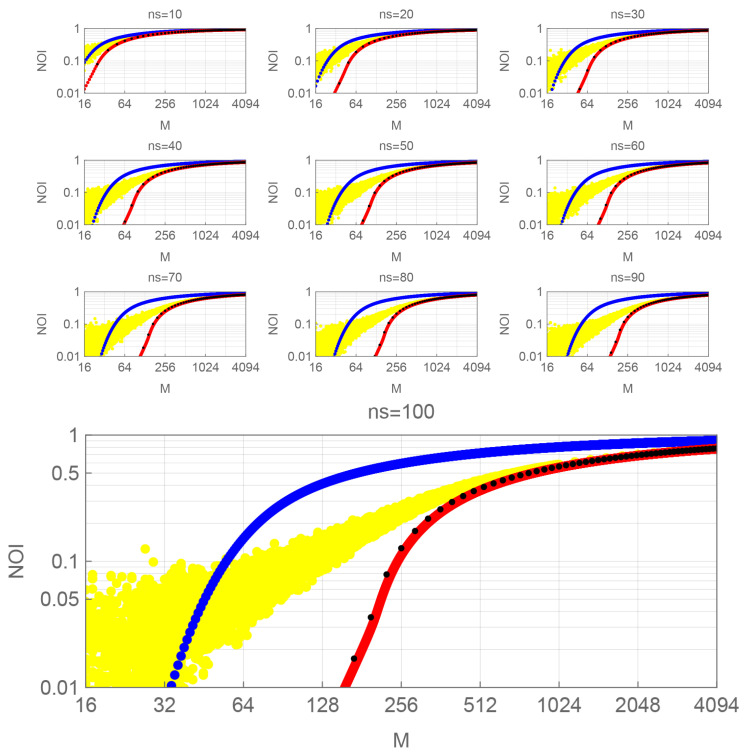
NOI vs. *M*. PSK: blue dots, QAM: black dots, SUN: red dots, random signals: yellow dots. Sub-parameter: ${\bar{n}}_{s}=10,20,30,40,50,60,70,80,90,100$.

Figure 2 shows the NOI performance of PSK, QAM, SUN, and random signals.

In each sub-figure for a fixed ${\bar{n}}_{s}$ shown in Figure 2, NOIs monotonically increase towards 1 in all cases as *M* increases. As ${\bar{n}}_{s}$ increases, the curves corresponding to each signal shift to the right. Furthermore, as *M* increases, PSK’s NOI increases more rapidly than QAM and SUN’s. The NOIs of QAM and SUN exhibit a similar value for each allowable *M*, but the NOI of QAM is slightly larger than that of SUN.

The NOIs of the random signals behave more like QAM and SUN than PSK at a large *M*. In other words, although QAM and SUN are designed signals, they behave similarly to randomly generated signals.

### 4.2. ${\bar{P}}_{c}$ vs. *M*

**Figure 3:**
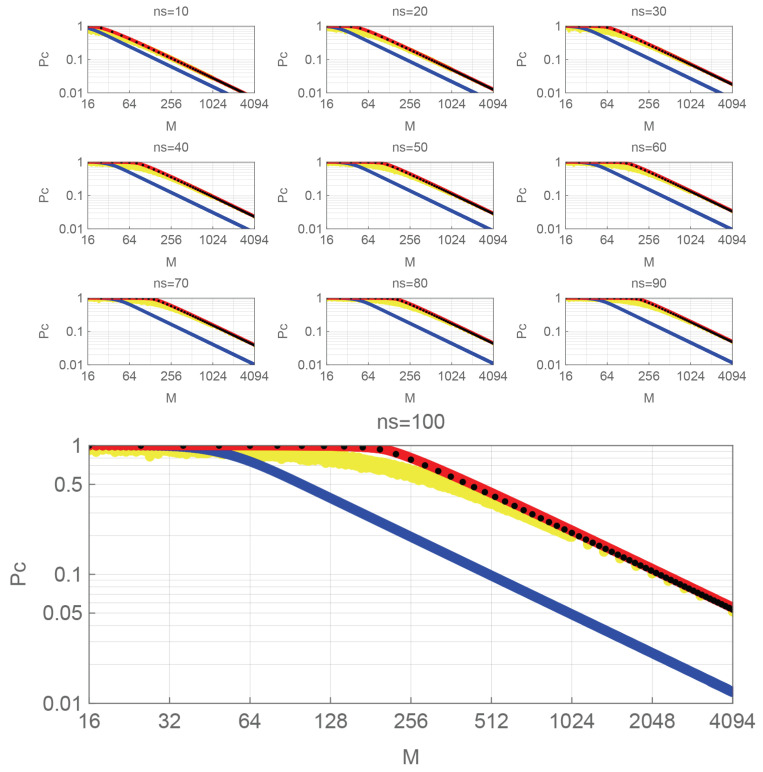
${\bar{P}}_{c}$ vs. *M*. PSK: blue dots, QAM: black dots, SUN: red dots, random signals: yellow dots. Sub-parameter: ${\bar{n}}_{s}=10,20,30,40,50,60,70,80,90,100$.

The sub-figures for each ${\bar{n}}_{s}$ shown in Figure 3 show that ${\bar{P}}_{c}$ decreases as *M* increases. From the viewpoint of the whole of Figure 3, the curves corresponding to each signal shift to the right as ${\bar{n}}_{s}$ increases.

Similar to the case of NOI, there are two groups: one consisting of PSK only and the other consisting of QAM and SUN. Comparing QAM and SUN, SUN’s ${\bar{P}}_{c}$ is slightly higher than QAM’s for the same *M*. It seems like a straight line when *M* is large because of a log-log plot. Based on this observation, the fitting curves can be obtained. For PSK, we have

(12) $${\bar{P}}_{c}^{\mathrm{psk}}\left({\bar{n}}_{s},M\right)\approx \frac{4.92{\bar{n}}_{s}^{0.504}}{M}$$

for sufficiently large *M* (about >500) and ${\bar{n}}_{s}$ of about 10∼100. Since ${\bar{n}}_{s}=\alpha_{0}^{2}$ for PSK, it can be simplified to the form $P_{c}^{\mathrm{psk}}\left(\alpha_{0},M\right)\approx 5\alpha_{0}/M$. For QAM and SUN, we have

(13) $${\bar{P}}_{c}^{\mathrm{qam}}\left({\bar{n}}_{s},M\right)\approx \frac{3.18{\bar{n}}_{s}^{0.904}}{M^{0.993}}\;\mathrm{and}\;{\bar{P}}_{c}^{\mathrm{sun}}\left({\bar{n}}_{s},M\right)\approx \frac{3.29{\bar{n}}_{s}^{0.914}}{M^{0.998}}.$$

## 5. Discussion

### 5.1. Relation Between the Non-Orthogonality and the Detection Probability

**Figure 4:**
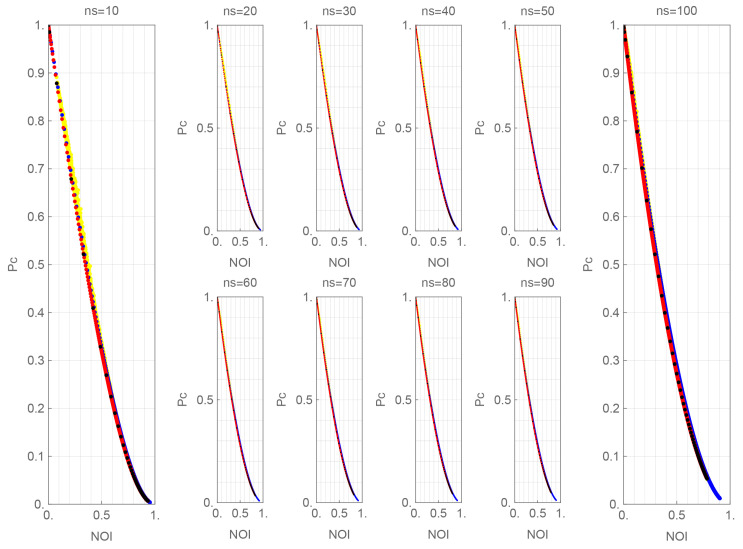
$P_{c}$ vs. NOI. PSK: blue dots, QAM: black dots, SUN: red dots, random signals: yellow dots. Sub-parameter: ${\bar{n}}_{s}=10,20,30,40,50,60,70,80,90,100$.

Figure 4 shows the relationship between non-orthogonality index NOI and detection probability ${\bar{P}}_{c}$, which is obtained by combining Figure 2 and Figure 3.

The PSK, QAM, and SUN curves in Figure 4 show a slightly downward-sloping curve. All the signals are overlaid on each graph, so they look like the same curve, but the curves are somewhat different. As ${\bar{n}}_{s}$ increases, the portion of the curve appearing in the mid-range of NOI shifts to the left as ${\bar{n}}_{s}$ increases for each signal.

To see those characteristics, we make approximation formulas in the following form:

(14) $${\bar{P}}_{c}\approx \exp\left[ax+bx^{2}+cx^{3}+dx^{4}+ex^{5}\right],\;\mathrm{and}\;x=NOI.$$

Table 2, Table 3 and Table 4 list the coefficients of the approximate formula for each signal valid for NOI values up to about 0.8∼0.95.

### 5.2. Comparison of Modulation Formats

In comparing the modulation formats, we found the following two main points:

(1) The numerical properties of PSK differ from those of QAM and SUN. As *M* increases, PSK’s NOI rises more rapidly than QAM and SUN, while PSK’s ${\bar{P}}_{c}$ decreases more quickly compared to QAM and SUN.

As shown in Figure 4, if a similar detection probability ${\bar{P}}_{c}$ is achieved for given ${\bar{n}}_{s}$, a similar NOI is needed for each. From Figure 2, the number of signals of QAM or SUN must be larger than that of PSK to obtain a similar NOI. This difference in the characteristics of NOI explains why the number *M* of signals for PSK-type and QAM-type quantum stream ciphers differs significantly.

(2) QAM and SUN exhibit a similar performance. As *M* increases, the behavior of the random signal approaches that of the QAM/SUN cases rather than the PSK case.

This result implies that QAM and SUN are “well-designed signals but behave closely to random signals.” In other words, this captures the characteristic that the signal points of QAM/SUN uniformly spread on the signal’s phase space. As QAM has been used in many concrete applications so far, it would be worthwhile to explore applications of SUN signals that have similar characteristics to QAM in the future.

## 6. Conclusions

The non-orthogonality of quadrature amplitude modulation (QAM) coherent state signals and a newly defined signal called the sunflower-like (SUN) coherent state signal were investigated numerically using the non-orthogonality index (NOI) and compared with phase shift keying (PSK) coherent state signals and randomly generated signals. The detection performance of these signals was also investigated.

As the number of signals increases for a given number of signal photons, the NOI of PSK increases faster than that of QAM and SUN, and the detection probability of PSK decreases faster than that of the others. PSK, QAM, and SUN show similar curves in the relationship between NOI and detection performance. In addition, QAM and SUN show similar performance in both NOI and detection probability, behaving similarly to randomly generated signals for a large number of signals. In other words, we observed that while QAM and SUN are well-defined, they behave similarly to random signals when the number of signals is large enough. Thus, QAM and SUN differ from PSK in terms of non-orthogonality and detection probability. Thus, the characteristics of QAM and SUN differ from those of PSK in terms of non-orthogonality and detection probability. Although QAM and SUN have similar characteristics, the main difference is that while QAM is limited in the number of signals, SUN can be defined in any number of signals. This flexibility in the number of possible signals will be an advantage of SUN in signal design for future applications in the era of AI and quantum computing.

In this study, the author proposed the SUN coherent state signal, which, unlike QAM, can be designed without a limit on the number of signals. Various other signal constellations can be defined with similar shapes. Analyzing these unexplored signal constellations will be an important topic for future research.

## Figures and Tables

**Table 1:** The average number of signal photons ${\bar{n}}_{s}$ (typical cases).

Modulation	*M* = 16	64	256	1024	4096
PSK	${\bar{n}}_{s}=\alpha_{0}^{2}$				
QAM	${\bar{n}}_{s}=10\beta_{0}^{2}$	$42\beta_{0}^{2}$	$170\beta_{0}^{2}$	$682\beta_{0}^{2}$	$2730\beta_{0}^{2}$
SUN	${\bar{n}}_{s}=8.5\gamma_{0}^{2}$	$32.5\gamma_{0}^{2}$	$128.5\gamma_{0}^{2}$	$512.5\gamma_{0}^{2}$	$2048.5\gamma_{0}^{2}$

**Table 2:** Approximation for PSK: ${\bar{P}}_{c}\approx \exp\left[ax+bx^{2}+cx^{3}+dx^{4}+ex^{5}\right]$, x=NOI.

${\bar{n}}_{s}$	*a*	*b*	*c*	*d*	*e*
10	−1.94	5.71	−29.7	48.5	−29.2
20	−1.92	4.28	−24.0	39.9	−24.7
30	−1.91	3.56	−21.1	35.4	−22.4
40	−1.91	3.10	−19.3	32.5	−20.8
50	−1.90	2.76	−17.9	30.3	−19.7
60	−1.90	2.50	−16.8	28.6	−18.8
70	−1.90	2.29	−16.0	27.2	−18.0
80	−1.89	2.12	−15.2	26.1	−17.4
90	−1.89	1.97	−14.6	25.1	−16.8
100	−1.89	1.84	−14.1	24.2	−16.4

**Table 3:** Approximation for QAM: ${\bar{P}}_{c}\approx \exp\left[ax+bx^{2}+cx^{3}+dx^{4}+ex^{5}\right]$, x=NOI.

${\bar{n}}_{s}$	*a*	*b*	*c*	*d*	*e*
10	−1.73	1.82	−14.0	24.6	−16.9
20	−1.81	1.09	−10.6	18.6	−13.4
30	−1.83	0.603	−8.46	14.9	−11.3
40	−1.85	0.328	−7.19	12.7	−9.96
50	−1.86	0.120	−6.23	11.0	−8.94
60	−1.86	−0.0394	−5.49	9.64	−8.13
70	−1.87	−0.165	−4.91	8.56	−7.47
80	−1.87	−0.264	−4.43	7.69	−6.92
90	−1.88	−0.345	−4.04	6.95	−6.46
100	−1.88	−0.414	−3.71	6.31	−6.05

**Table 4:** Approximation for SUN: ${\bar{P}}_{c}\approx \exp\left[ax+bx^{2}+cx^{3}+dx^{4}+ex^{5}\right]$, x=NOI.

${\bar{n}}_{s}$	*a*	*b*	*c*	*d*	*e*
10	−1.91	3.91	−21.8	36.0	−22.6
20	−1.90	2.19	−14.8	24.8	−16.6
30	−1.89	1.35	−11.3	19.2	−13.6
40	−1.89	0.842	−9.15	15.7	−11.6
50	−1.89	0.497	−7.67	13.2	−10.2
60	−1.89	0.249	−6.59	11.4	−9.09
70	−1.89	0.0604	−5.76	9.92	−8.24
80	−1.89	−0.0867	−5.11	8.77	−7.55
90	−1.89	−0.204	−4.57	7.82	−6.97
100	−1.89	−0.300	−4.13	7.02	−6.48

## Data Availability

The original contributions presented in this study are included in the article. Further inquiries can be directed to the corresponding author.

## Abbreviations

The following abbreviations are used in this manuscript:

NOI	Non-orthogonality index
SRM	Square-root measurement
PSK	Phase shift keying
QAM	Quadrature amplitude modulation
SUN	Sunflower (sunflower-like modulation)
